# Functional Reconstitution of HlyB, a Type I Secretion ABC Transporter, in Saposin-A Nanoparticles

**DOI:** 10.1038/s41598-019-44812-0

**Published:** 2019-06-10

**Authors:** Kerstin Kanonenberg, Sander H. J. Smits, Lutz Schmitt

**Affiliations:** 1 Institute of Biochemistry, Heinrich Heine University, Universitaetsstr. 1, 40225 Duesseldorf, Germany; 2 Present Address: Université de Lyon, CNRS, UMR5086 “Molecular Biology and Structural Biochemistry”, IBCP, Lyon, France

**Keywords:** Proteins, Membrane lipids

## Abstract

Type I secretion systems (T1SS) are ubiquitous transport machineries in Gram-negative bacteria. They comprise a relatively simple assembly of three membrane-localised proteins: an inner-membrane complex composed of an ABC transporter and a membrane fusion protein, and a TolC-like outer membrane component. T1SS transport a wide variety of substrates with broad functional diversity. The ABC transporter hemolysin B (HlyB), for example, is part of the hemolysin A-T1SS in *Escherichia coli*. In contrast to canonical ABC transporters, an accessory domain, a C39 peptidase-like domain (CLD), is located at the N-terminus of HlyB and is essential for secretion. In this study, we have established an optimised purification protocol for HlyB and the subsequent reconstitution employing the saposin-nanoparticle system. We point out the negative influence of free detergent on the basal ATPase activity of HlyB, studied the influence of a lysolipid or lipid matrix on activity and present functional studies with the full-length substrate proHlyA in its folded and unfolded states, which both have a stimulatory effect on the ATPase activity.

## Introduction

Secretion systems are essential means for prokaryotic organisms to deliver a large set of nascent proteins, including virulence factors, from the cytoplasm to the extracellular environment. In Gram-negative bacteria, secretion of molecules needs to be achieved across both membranes. Thus, many secretory pathways have evolved that export their substrate, in some cases with a periplasmic intermediate, while the prototype type I secretion system (T1SS), however, has no such intermediate^1^.

One of the most prominent members of the T1SS is the hemolysin A (HlyA) secretion system from *Escherichia coli* (*E. coli*)^2^. The substrate, the hemolytically active exotoxin HlyA, is secreted unfolded in one step, without periplasmic intermediate, across both membranes^3–5^. T1SS generally comprise a relatively simple assembly of an ABC transporter and a membrane fusion protein in the inner membrane, and a TolC-like outer membrane protein. In the case of HlyA-T1SS, these are hemolysin B (HlyB), hemolysin D (HlyD) and TolC, respectively^6,7^. While HlyB and HlyD form a complex in the inner membrane, TolC, which is also involved in many other export processes, is only recruited upon substrate recognition in the cytosol^8^.

The substrate HlyA contains a secretion signal that has been located to the last 50–60 C-terminal amino acids^4,9,10^. Additionally, HlyA belongs to the family of RTX-proteins (“repeats in toxin”), whose characteristic is the presence of a variable number of nonapeptide repeats (RTX-domains) that bind calcium ions and trigger folding of the protein in the extracellular space^5,11,12^. The acylation of two lysine-residues activate the toxin prior to secretion^13^. Secretion takes place C-terminus first^5^ leaving open for debate how substrates of several 100 kDa in size are kept unfolded in the cytosol until secretion is initiated. The secretion rate was determined to be 16 amino acids per second per transporter^14^.

The ABC transporter HlyB fuels the secretion process by ATP-hydrolysis. Interaction between the nucleotide binding domain (NBD) of HlyB and the substrate HlyA has been demonstrated, suggesting a supplementary function in addition to providing energy for export^15^. The mechanism of ATP-hydrolysis of the isolated HlyB-NBD has been investigated in detail^16–18^. More importantly, HlyB contains an additional cytosolic domain at its N-terminus that is essential for secretion^19,20^. A similar N-terminal domain is known for bacteriocin exporters, which contain a C39-peptidase domain that cleaves the N-terminal signal peptide of the substrate prior to export^21^. Despite its identical tertiary structure compared to C39 peptidase domains, the additional domain of HlyB does not catalyse a proteolytic reaction. This is due to a corrupted and inactivated catalytic triad^20,22^. Subsequently, the term C39-peptidase-like domain (CLD) was coined^20^. The unfolded substrate interacts with the CLD, independently of its C-terminal secretion signal^20^. Thus, receptor or chaperone-like activity has been proposed for the CLD, but its precise function and mechanism are not yet understood.

The bottleneck in studying membrane proteins *in vitro* is often related to the requirement to purify membrane proteins to high purity and homogeneity. When using detergents for their extraction from membranes, membrane proteins are pulled out of their natural environment. Furthermore, the presence of monomeric detergent can result in binding to non-native locations on the membrane protein, for example between helices or in hydrophilic areas^23–25^. This may have a negative effect on the activity of a membrane protein in detergent solution^26^. To overcome this issue, membrane proteins can be reconstituted into artificial lipid bilayer systems, such as liposomes or nanodiscs. Reconstitution into liposomes results in a two-compartment system, which is a great advantage in studying transport processes^27^, and a 13-fold higher ATPase activity of proteoliposome-reconstituted ABC transporter BmrA has been reported in comparison to detergent-solubilised preparations^28–30^. Liposomes can be prepared with defined lipid compositions, which also makes them a suitable tool for studying the influence of lipids on a membrane protein. However, due to their large size, liposomes are not suitable for downstream purification processes, such as size exclusion chromatography (SEC).

The nanodisc system offers the advantage that the membrane protein is incorporated into a small yet water-soluble particle, where the lipid composition may be tailored^31–33^. Nanodiscs of well-defined dimensions have facilitated the handling of reconstituted protein and have been shown to be a suitable tool for diverse applications in biophysics or structural biology^33^. A very recent, additional development is a saposin-A derived lipoprotein nanoparticle system^34^. *In vivo*, proteins belonging to the saposin-family modulate the lipid composition of lysosome membranes^35,36^. Their lipid binding properties, paired with their composition out of amphipathic helices containing six disulphide bridges, result in the formation of very stable and uniform nanoparticles^34^. Surprisingly, even in the presence of detergent, saposin-A forms dimeric structures incorporating a small bilayer of detergent molecules^37^. Purified saposin-A can be used as carriers of small lipid bilayers with embedded membrane proteins^34^. An advantage is the easy purification of saposin-A, the rapid process of reconstitution, and the very practicable separation of “full” and “empty” nanoparticles^34,38–40^. The focus of studies applying the saposin-A system so far has been on structural biology, showing its suitability for cryo EM^38^, solution NMR^39^, or SAXS^40^, while functional studies on saposin-reconstituted proteins are lacking.

Here, we report the functional reconstitution of the *E. coli* ABC transporter HlyB from the HlyA-T1SS into saposin-based nanoparticles. We show that the system is suitable for the functional characterisation of the ABC transporter. Equally important, reconstitution in nanoparticles was achieved not only in the presence of lipids, but for the first time also in the presence of detergent-like lysolipids. The functional data of reconstituted HlyB revealed important differences from those obtained for detergent-solubilised HlyB, especially in comparison to a truncated mutant lacking the CLD^41^, and highlights the negative impact of free detergent in buffer solution on the activity of HlyB. By characterising the T1SS ABC transporter, we are aiming to understand the function of the different domains during the secretion process.

## Materials and Methods

### Cloning of the plasmid pBADHisHlyB-D551A

The plasmid pBADHisHlyB-D551A was created by introducing a point mutation into pBADHisHlyB^41^. For this, the plasmid was amplified with Pfu-polymerase (NEB) using the primers GGTTGTGTTGCAGGCAAATGTGCTGCTTAATCG (forward) and CGATTAAGCAGCACATTTGCCTGCAACACAACC (reverse). Methylated bacterial DNA was digested using DpnI (NEB) and the PCR product was used to transform *E. coli* XL-1 blue cells. The introduction of the point mutation was confirmed by sequencing.

### Construction of the expression strain for HlyB by genomic modification

To delete *acrAB* from *E. coli* C41(DE3)∆*ompF* strain, the lambda-red recombinase system was employed, following published protocols^42^. Briefly, the plasmid pKD4 carrying a kanamycin-resistance cassette was amplified using the overhang primers ACTTTTGACCATTGACCAATTTGAAATCGGACACTCGAGGTTTACATATGAGTAGGCTGGAGCTGCTTC (forward) and TTACGCGGCCTTAGTGATTACACGTTGTATCAATGATGATCGACAGTATGATGGGAATTAGCCATGGTCC (reverse). The insertion of the kanamycin-resistance cassette using the plasmid pKD46 and its deletion with plasmid pCP20 were confirmed by PCR and subsequent sequencing using the primers CACATCGAGGATGTGTTG (forward) and GCCCTCTCGTTTGTTAG (reverse).

### Overexpression and purification of HlyB

*E. coli* C41(DE3)*∆ompF∆acrAB* cells were transformed with pBADHisHlyB, pBADHisHlyB∆CLD, pBADHisHlyB-H622A^41^ or pBADHisHlyB-D551A plasmids and selected on LB-agar plates containing 100 µg/mL ampicillin. All HlyB variants were overexpressed following published protocols^41^.

Membranes were isolated by a two-step centrifugation procedure. First, cells were resuspended in buffer P (50 mM NaH_2_PO_4_ pH 8, 300 mM NaCl) and lysed by passing three times through a cell disruptor (Microfluidizer M-110L, Microfluidics) at 1.5 kbar. Undisrupted cells and cell debris were removed by low-spin centrifugation at 18,000 × g for 30 min at 4 °C. Membranes were collected from the supernatant by ultra-centrifugation at 150,000 × g for 90 min at 4 °C. Membrane pellets were homogenised in buffer P supplemented with 10% (v/v) glycerol and stored at −80 °C.

For the purification of HlyB, membranes of 0.5 L cell culture were diluted with buffer P to a protein concentration of 10 mg/mL and solubilised with 0.5% (w/v) fos-choline 14 for 1 h at 8 °C. Solubilised membranes were filtered (0.45 µm), diluted two-fold using buffer P supplemented with 2 mM imidazole and loaded on Zn^2+^-charged immobilised metal-ion affinity chromatography (IMAC) column (5 mL HiTrap Chelating HP, GE Healthcare). The column was washed with 8 mL of buffer P including 0.015% (w/v) DDM and 2 mM imidazole. Non-specifically bound proteins were removed by washing with 18 mL buffer P supplemented with 0.015% (w/v) DDM and 40 mM imidazole. HlyB was eluted with buffer P containing 0.015% (w/v) DDM and 25 mM EDTA.

### Expression and purification of proHlyA from inclusion bodies

*E. coli* BL21(DE3) cells were transformed with pSU-HlyA^13^ and exposed on selective LB-agar plates containing 100 µg/mL ampicillin. An overnight culture with 2YT medium and 100 µg/mL ampicillin was inoculated with a single colony and incubated for 15 h at 200 rpm, 37 °C. The main cultures were grown in 5 L-baffled flasks, containing 1 L of selective 2YT medium with 100 µg/mL ampicillin. Main cultures were inoculated from the overnight culture to OD_600_ of 0.1 and grown at 37 °C, 200 rpm to OD_600_ of 0.6. ProHlyA expression was induced by adding IPTG to a final concentration of 1 mM. Incubation was continued for 4 h and cells were harvested by centrifugation.

For proHlyA purification, cells were resuspended in buffer A (50 mM HEPES pH 7.4, 150 mM NaCl, 10% (w/v) glycerol, 0.05% (w/v) NaN_3_) and lysed by passing three times through the cell disruptor at 1.5 kbar. Inclusion bodies were collected by centrifugation at 18,000 × g for 30 min. The pellets were washed and centrifuged successively in (1) buffer W1 (50 mM HEPES, pH 7.4, 50 mM EDTA, 1% (w/v) Triton X-100, 0.05% (w/v) NaN_3_) and (2) buffer W2 (50 mM HEPES, pH 7.4, 1 mM EDTA, 1 M NaCl, 0.05% (w/v) NaN_3_). The pellet was solubilised overnight in buffer S (20 mM HEPES pH 7.4, 20 mM NaCl, 6 M urea). Insoluble material was removed by ultra-centrifugation (150,000 × g, 30 min, 4 °C) and the urea-solubilised inclusion bodies were stored at −80 °C.

### Expression and purification of saposin-A

Overexpression and purification of saposin-A was performed following the described protocol^34^ with minor modifications. Briefly, *E. coli* Rosetta-gami-2 (DE3) cells (Novagen) were transformed with pSapA plasmid^34^ and grown on selective LB-agar plates containing 25 µg/mL chloramphenicol and 30 µg/mL kanamycin. An overnight culture of 2YT medium supplemented with 25 µg/mL chloramphenicol and 30 µg/mL kanamycin was inoculated with a single colony and shaken at 200 rpm, 37 °C for 20 h. Main cultures of 1 L 2YT medium in 5 L-baffled flasks, supplemented with 30 µg/mL kanamycin, were inoculated to OD_600_ of 0.1 using the overnight culture. Cells were grown to OD_600_ of 1 (approximately 7 h) at 200 rpm, 37 °C. Protein expression was induced by adding IPTG to a final concentration of 0.7 mM and continued for 3 h at 200 rpm, 37 °C.

For the purification of Saposin-A, cells were resuspended in buffer A (20 mM HEPES, pH 7.5, 150 mM NaCl, 20 mM imidazole) and lysed by passing three times through the cell disruptor at 1.5 kbar. Lysed cells were centrifuged at 26,000 × g for 30 min and the supernatant was heated to 85 °C for 10 min, followed by a second centrifugation step at 26,000 × g for 30 min. The supernatant was applied to a Ni^2+^-charged IMAC column (5 mL HiTrap Chelating HP, GE Healthcare). The column was washed with 15 column volumes (CV) using buffer A, followed by 10 CV buffer A supplemented with 40 mM imidazole. Elution was performed with buffer A containing 400 mM imidazole. The eluted protein was concentrated to a final volume of 5 mL (Amicon Ultra-15, MWCO = 10,000 Da, Merck/Millipore), centrifuged at 100,000 × g for 10 min and subjected to SEC on HiLoad 16/600 Superdex 200 pg column (GE Healthcare) in buffer B (20 mM HEPES pH 7.5, 150 mM NaCl). The purified saposin-A was stored at a concentration of 1.2 mg/mL at −20 °C.

### Reconstitution of HlyB into saposin-A nanoparticles

Reconstitution procedures were adapted from^34^. Either 60 µL of 5 mg/mL DOPC in 100 mM HEPES, pH 8, 250 mM NaCl, 1% (w/v) DDM or 40 µL of 5 mg/mL LPC in 100 mM HEPES, pH 8, 250 mM NaCl, 1% (w/v) DDM were heated to 30 °C for 5 min. 60 µL IMAC-purified HlyB (1.4–1.6 mg/mL) were added (protein :  lipid ratio = 1 :  390) and incubated at 30 °C for 5 min. 60 µL saposin-A (1.2 mg/mL) were added, followed by another incubation at 30 °C for 5 min. After adding 500 µL buffer H (100 mM HEPES, pH 8, 250 mM NaCl), samples were incubated at room/ambient temperature for 5 min. 5 aliquots were pooled and concentrated to a final volume of 1 mL (Amicon Ultra-15, MWCO = 100,000 Da, Merck/Millipore). Protein was subjected to SEC in buffer H on a Superose 6 Increase 10/300GL column (GE Healthcare).

### ATPase assays

Determination of the amount of free phosphate was carried out to quantify the hydrolytic activity of HlyB^43^. For basal ATPase activity, 15 µL of HlyB in buffer H were supplemented with 5 µL of 50 mM MgCl_2_. Control reactions did not contain MgCl_2_. Reactions were started by adding ATP to final concentrations ranging from 0 to 8 mM (total reaction volume 25 µL), and incubated for 40 min at 25 °C.

To measure the modulation of the ATPase activity by substrate, folded and unfolded proHlyA was added to the assay in final concentrations ranging from 0–10 µM. Folded proHlyA was prepared in buffer H supplemented with 10 mM CaCl_2_, unfolded proHlyA in buffer H supplemented with 4 M urea and 10 mM EDTA. Buffers were exchanged to buffer H supplemented with 2 mM CaCl_2_ or 4 M urea, respectively.

10 µL of HlyB were supplemented with 5 µL of 50 mM MgCl_2_ and 5 µL of proHlyA. Reactions were started by adding 5 µL of 20 mM ATP and incubated for 30 min (stimulatory effects) or 60 min (inhibitory effects) at 25 °C.

The effects of free LPC on HlyB ATPase activity were determined by adding 5 µL of LPC to the assay in final concentrations ranging from 0.1 µM to 1 mM. Assays were conducted as described for proHlyA.

Reactions were stopped by transferring the reaction volume (25 µL) into 175 µL 10 mM H_2_SO_4_. The free phosphate concentration was determined by adding 50 µL of staining solution (0.096% (w/v) malachite green, 1.48% (w/v) ammonium molybdate, 0.173% (w/v) Tween-20 in 2.36 M H_2_SO_4_) and incubated for 8 min at room/ambient temperature. Quantification was performed by measuring the absorbance at 595 nm (iMark Microplate Reader, Bio Rad). Data points were fitted using GraphPad Prism 8 Software (GraphPad).

### Determination of kinetic parameters of ATPase activity

The experimental data sets were fitted to one of the following equations:

Equation 1, the Hill equation, is:

1 $$v=\frac{{v}_{max}{[S]}^{h}}{{{K}_{0.5}}^{h}+{[S]}^{h}}$$

In Eq. 1, v corresponds to the enzyme velocity as a function of the substrate concentration [S], v_max_ is the maximum enzyme velocity, h is the Hill coefficient, K_0.5_ is the substrate concentration at half-maximum enzyme velocity.

Equation 2, the Michaelis-Menten equation, with substrate inhibition is:

2 $$v=\frac{{v}_{max}[S]}{{K}_{m}+[S](1+\frac{[S]}{{K}_{i}})}$$

Here, K_m_ is the Michaelis-Menten constant and K_i_ the dissociation constant for substrate binding to the enzyme. It is assumed that two substrate molecules can bind to the enzyme, which results in a stimulatory or inhibitory effect, respectively.

Equation 3, which assumes two independent binding sites, is:

3 $$v=\frac{{K}_{1}{K}_{2}{v}_{0}+{K}_{2}{v}_{max}+{v}_{min}{[S]}^{2}}{{K}_{1}{K}_{2}+{K}_{2}[S]+{[S]}^{2}}$$

Here, v_min_ is the minimum enzyme velocity, K_1_ the substrate concentration at half-maximum stimulation, K_2_ the substrate concentration at half-maximum inhibition. Equation 3 was adapted from^41^.

Statistical analysis (extra-sum-of-squares F-test) was performed using the software package Prism 8 (Graphpad).

### Statistical analysis

All statistical analyses were performed using the software package Prism 8 (Graphpad). For the comparison of two samples, the t-test was employed. When comparing more than two samples, a one-way ANOVA test was used.

## Results

### Expression and purification of the ABC transporter HlyB using a new expression strain, *E. coli* C41(DE3)∆ompF∆acrAB

The deletion of *ompF* and *acrAB* from the genome of *E. coli* C41(DE3) was found to substantially increase the yield and the purity of isolated recombinant HlyB.

The use of fos-choline 14 (FC-14) as a suitable detergent for the solubilisation of HlyB was adopted from a previous study^41^. The detergent was exchanged to 0.015% (w/v) DDM or 0.003% (w/v) LMNG while HlyB was immobilised on the IMAC column. Detergent exchange is more efficient while the protein is bound to IMAC resin than during SEC, as the washing can be largely extended^44^. Furthermore, empty detergent micelles are of considerable size and often migrate through the SEC column close to or even with the protein^45^. Especially fos-cholines, as used in this study, have a very small cmc and are difficult to exchange by SEC alone.

**Figure 1:**
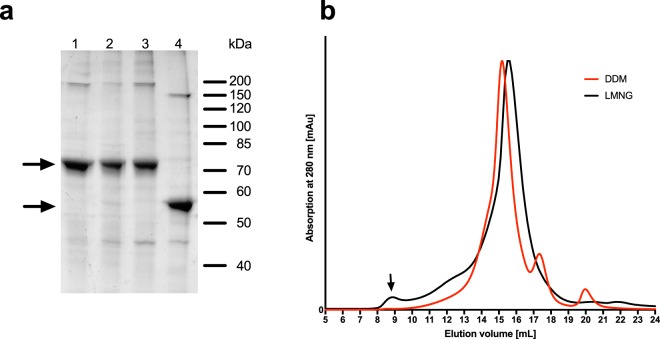
Purification of HlyB. (**a**) – SDS-PAGE of purified HlyB (1), HlyB-H622A (2), HlyB-D551A (3) and HlyB∆CLD (4), stained with CBB. The arrows indicate the monomers of HlyB, HlyB H622A and HlyB D551A (theoretical molecular weight 82 kDa, but it migrates at approximately 70 kDa) and HlyB∆CLD (65 kDa, migration at 55 kDa)^62^. (**b**) – SEC of purified HlyB (Superose 6 10/300GL) in LMNG (black line) and DDM (red line). The arrow indicates the void volume of the column.

Both DDM and LMNG yielded approximately 6 mg of pure and homogeneous HlyB per litre of bacterial cell culture (Fig. 1). The homogeneity after purification was assessed by SEC and the elution profile was comparable for DDM and LMNG-purified protein (Fig. 1b). Equivalent amounts of protein (6–8 mg per litre of cell culture) were obtained for HlyB, HlyB∆CLD, HlyB-H622 A and HlyB-D551A.

### Reconstitution of HlyB into saposin-A lipoprotein-nanoparticles

In the crystal structure of the saposin-A dimer a bilayer-like assembly of detergent molecules trapped between the protein protomers was observed^37^. To assess the influence of the density of the lipid bilayer around HlyB on its functional properties we prepared saposin-A nanoparticles using DOPC (two fatty acid tails, high-density lipid bilayer with low curvature) or LPC (one fatty acid tail, low-density lipid bilayer with high positive curvature)^46,47^.

While general guidelines for saposin nanoparticle reconstitution have been previously described^34^, we set out to optimise the ratio HlyB : saposin-A  :  lipid for achieving high yields of reconstituted HlyB. It is a common procedure to add a large excess of membrane scaffold protein to the reconstitution mixture^34,38^, as it has been described before also for other reconstitution systems such as nanodiscs^48,49^.

**Figure 2:**
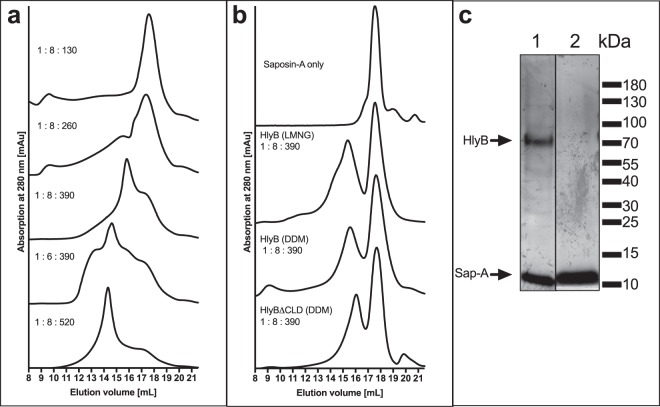
Reconstitution of HlyB and HlyB∆CLD into saposin-A nanoparticles. The optimum molar ratio was identified to be 1 :  8 :  390 (HlyB :  saposin-A  :  lipids) for DOPC as well as for LPC, which results in homogeneous peaks. (**a**) Optimisation of the reconstitution into DOPC-saposin-A nanoparticles. Ratios were molar ratios of HlyB :  saposin-A  :  DOPC. (**b**) Optimisation of the reconstitution of HlyB into LPC-saposin-A nanoparticles. The detergent used for the purification of HlyB is indicated in brackets. The use of LMNG-purified HlyB resulted in preparations of lower homogeneity. Ratios are molar ratios of HlyB :  saposin-A  :  LPC. (**c**) SDS-PAGE of reconstituted DDM-purified HlyB into LPC-particles after SEC on a 3–20% gradient silver-stained gel. Bands were visible for HlyB at 70 kDa and saposin-A at 10 kDa (both indicated by arrows). (1) Peak at elution volume 15.5 mL (reconstituted HlyB), (2) peak at elution volume 17.8 mL (“empty” saposin-A particles).

To optimise the reconstitution using DOPC, the amount of HlyB was kept constant and the amounts of lipids and saposin-A were varied, and the reconstituted products were analysed by SEC (Fig. 2a) in order to evaluate the amounts of reconstituted ABC transporter and assess its homogeneity at different ratios. The optimum HlyB :   saposin-A :  DOPC ratio was found to be 1 :  8 :  390 as a homogeneous sample of reconstituted protein was obtained, characterised by a homogeneous peak of the reconstituted ABC transporter at a retention volume of 15.5 mL. The peak eluting at 17–18 mL corresponds to “empty” saposin-A particles. Increasing amounts of DOPC resulted in the formation of inhomogeneous preparations (e.g. 1 :  6 :  390) or larger species of particles, which presumably contained more than one reconstituted ABC transporter (e.g. 1 :  8 :  520). Lower amounts of lipids reduced the amount of reconstituted HlyB (e.g. 1 :  8 :  260).

The reconstitution of HlyB into saposin-A nanoparticles using LPC was equally optimised (Fig. 2b). Strikingly, the optimal HlyB :  saposin-A  :  LPC molar ratio was also 1 :  8 :  390 (Fig. 2b), suggesting that the detergent and lipid packing within the nanoparticles was largely determined by the head groups, and was comparable for DOPC and LPC.

Furthermore, for successful reconstitution the choice of detergent during the purification of HlyB also needed optimisation.

To our surprise, the optimal ratio of 1 :  8 :  390 resulted in a homogeneous preparation of DDM-purified HlyB, with two distinctively separated peaks of reconstituted HlyB and “empty” saposin-A particles (Fig. 2b,c). The use of LMNG-purified HlyB but otherwise identical reconstitution conditions resulted in less homogeneous preparations of HlyB-containing particles (Fig. 2b), even though HlyB was equally homogeneous in both detergents after purification (Fig. 1b).

The reconstitutions of HlyB∆CLD, HlyB-D551A and HlyB-H622A produced comparable results.

### Basal ATPase activity of reconstituted HlyB

**Figure 3:**
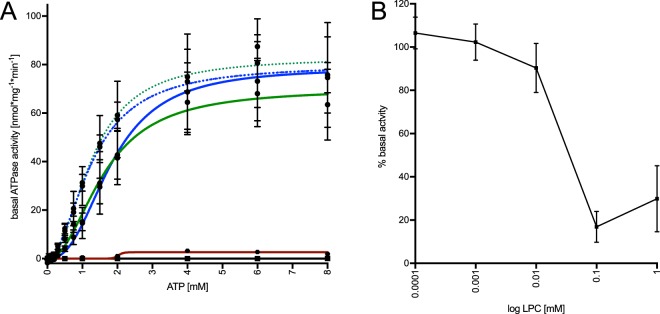
(**A**) Kinetic measurements of the basal ATPase activity of HlyB in LPC (green solid line) and DOPC (green dashed line), HlyB∆CLD in LPC (blue solid line) and DOPC (blue dashed line), HlyB D551A in LPC (red solid line) and HlyB H622A in LPC (black line). Error bars represent SEM of a minimum of two replicates. (**B**) Influence of free LPC on the basal ATPase activity of DOPC-reconstituted HlyB. As the free LPC-concentration increases, the basal ATPase activity of HlyB is inhibited.

The basal ATPase activity of reconstituted HlyB was measured by quantifying the release of inorganic phosphate from the hydrolysis of ATP^43^. The ATP concentration was varied from 0 to 8 mM, while the concentrations of HlyB and MgCl_2_ were kept constant. The ATPase activities of HlyB, HlyB∆CLD, HlyB-D551A and HlyB-H622A were assessed in LPC-saposin-A nanoparticles. The results are summarised in Fig. 3A (solid lines).

HlyB-H622A, a HlyB mutant defective in ATP hydrolysis^18,41^, showed no ATP hydrolysis under the experimental conditions and served as a negative control.

The data for HlyB and HlyB∆CLD displayed a non-linear behaviour and were fitted using the Hill-equation. The data showed a clear sigmoidal fit and differ significantly from the Michaelis-Menten model, which was shown by performing an extra-sum-of-squares F-test (Supplementary Fig. 3 and Supplementary Table 1). This behaviour has been described before for the isolated HlyB-NBD^8^ as well as full-length HlyB in detergent solution^42^. Within the standard errors, the ATPase kinetic parameters of both proteins, HlyB and HlyB∆CLD, were equal, with Hill coefficients of 2.1 ± 0.6 and 2.5 ± 0.8, respectively. The v_max_ and K_0.5_ values for HlyB and HlyB∆CLD were 70 ± 7 nmol mg^−1^ min^−1^, 1.6 ± 0.3 mM (HlyB), and 79 ± 8 nmol mg^−1^ min^−1^, 1.8 ± 0.3 mM (HlyB∆CLD), respectively. The kinetic parameters of the basal ATPase activity are summarised in Table 1.

**Table 1:** Summary of the kinetic parameters of HlyB and HlyB∆CLD ± SEM of minimum two replicates.

	K_0.5_ [mM]	v_max_ [nmol min^−1^ mg^−1^]	h	k_cat_ [min^−1^]
HlyB (LPC)	1.6 ± 0.3	70.0 ± 6. 6	2.1 ± 0.6	5.8 ± 0.5
HlyB (DOPC)	1.3 ± 0.1	82.9 ± 1.9	2.1 ± 0.2	6.8 ± 0.2
HlyB∆CLD (LPC)	1.8 ± 0.3	78.7 ± 7.6	2.5 ± 0.8	5.3 ± 0.5
HlyB∆CLD (DOPC)	1.3 ± 0.1	79.9 ± 4.3	1.9 ± 0.3	5.4 ± 0.3

The aspartate residue 551 was previously suggested to play an important role in the communication between the two ATP-binding domains and the asymmetric phosphate release. This resulted in a reduced ATPase activity and loss of cooperativity, as evident from studies of the isolated NBD^50^. We analysed this mutation in the context of the full-length ABC transporter and observed that HlyB-D551A showed no hydrolytic activity under the experimental conditions (Fig. 3A). Next, we measured the basal ATPase activity of HlyB and HlyB∆CLD in DOPC-saposin-A particles (Fig. 3A, dashed lines). The kinetic parameters were equal within the standard errors to those in LPC-saposin-A particles and are summarised in Table 1.

We assessed the impact of free detergent on the activity of HlyB by adding increasing concentrations of LPC to the assay using DOPC-reconstituted protein. LPC concentrations exceeding 0.01 mM significantly reduced the basal ATPase activity of HlyB to approximately 10% of the original ATPase activity (Fig. 3B). Thus, we conclude that the ATPase activity of HlyB is strongly affected by the presence of free detergent and the incorporation of the transporter into micelles. This effect can be prevented by reconstitution into saposin-A particles, independent of the nature of the bilayer (DOPC or LPC).

### Folded and unfolded substrates modulate the ATPase activity of HlyB in different ways

Differences between DOPC- and LPC-reconstituted HlyB became apparent in functional assays with the full-length substrate proHlyA in its unfolded and folded states.

In contrast to other studies conducted with detergent-purified HlyB where a shortened version of the natural substrate was used in the ATPase assays^41^, we aimed to employ the full-length, yet hemolytically inactive precursor form of HlyA, proHlyA. Hemolytic activity of HlyA requires two acylations at K563 and K689 and these are absent in proHlyA. Importantly, these acylations are irrelevant for the secretion process and the secretion rate^13^.

A particular challenge of working with the full-length substrate is its size (110 kDa) and its tendency to aggregate already at low concentrations when kept unfolded in the Ca^2+^-unbound state. In order to mimic the *in vivo* situation, where unfolded substrate interacts with the ABC transporter, proHlyA was prepared in stock-solutions containing 4 M urea, which resulted in a final urea concentration of 0.8 M in the ATPase assay, but the basal ATPase activity of HlyB was not influenced by the final urea concentration (Supplementary Fig. 4). Folded proHlyA was prepared by adding Ca^2+^ to and removing urea from the buffer. Measurements with both types of proHlyA, folded and unfolded, were conducted at substrate concentrations ranging from 0 to 10 µM, respectively. ATPase activity was measured at uniform ATP, MgCl_2_ and HlyB concentrations. The chosen concentration of ATP of 4 mM was approximately 3-fold above the K_0.5_ to ensure quantitative ATP saturation of HlyB. The observed effects thus resulted from interactions of the substrate with the ABC transporter, since no ATPase activity of folded or unfolded proHlyA alone was observed.

**Figure 4:**
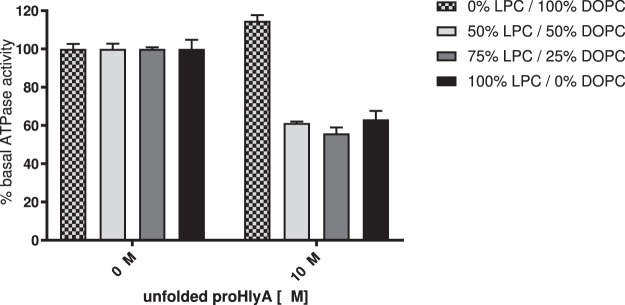
Stimulation of the basal ATPase activity of HlyB by 10 µM of the substrate proHlyA. HlyB was reconstituted with different DOPC/LPC mixtures, as indicated in the figure legend. No stimulation by the substrate was observed in the absence of LPC in the nanoparticles.

No interaction of the unfolded substrate with HlyB was observed in DOPC-particles, reflected by the absence of any stimulation of the basal-level ATPase activity (Fig. 4). We prepared mixed DOPC-LPC-particles by using mixtures containing 0–100% (molar ratio) LPC. When LPC/DOPC mixtures or LPC alone were used for reconstitution, the addition of unfolded proHlyA resulted in an inhibition of the basal ATPase activity of approximately 40% (Fig. 4). Thus, further kinetic measurements in the presence of substrate were performed with LPC-reconstituted HlyB.

**Figure 5:**
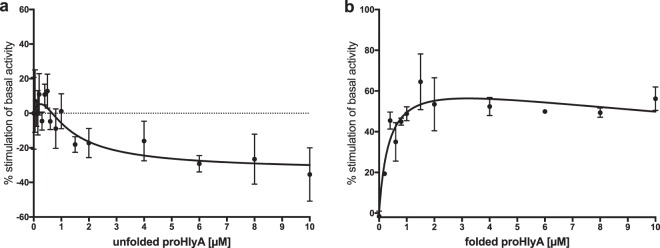
Kinetic measurements of HlyB in LPC-saposin-A particles in the presence of (**a**) unfolded proHlyA and (**b**) folded proHlyA. The dashed line in (**a**) reflects the basal ATPase activity in the absence of substrate. Error bars represent SEM of a minimum of three biological replicates.

Unfolded and folded proHlyA were found to interact differently with HlyB reconstituted in LPC-particles, resulting in opposing modulating effects. Adding the unfolded substrate resulted in an inhibition of the basal ATPase activity by approximately 30%, while the folded substrate resulted in a stimulation of ATP hydrolysis by 60% (Fig. 5). As (pro)HlyA remains unfolded in the cytosol until secretion is initiated, folded substrate does not constitute a natural substrate of HlyB and the stimulating effect was an unexpected finding.

### The N-terminal CLD of HlyB is required for a specific interaction with unfolded substrate

**Figure 6:**
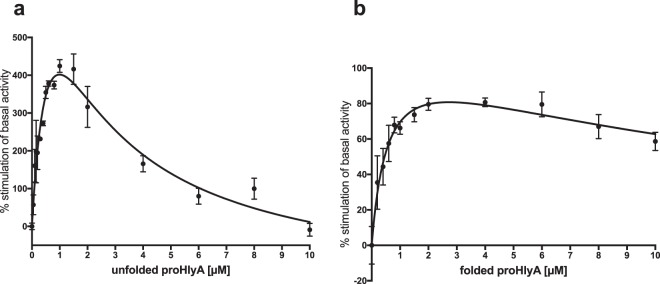
Kinetic measurements of HlyB∆CLD in LPC-saposin-A particles in the presence of (**a**) unfolded proHlyA and (**b**) folded proHlyA. Error bars represent SEM of minimum two biological replicates.

As the cytoplasm-exposed CLD of HlyB has been speculated to interact with the substrate prior to translocation, we employed the truncated mutant HlyB∆CLD to investigate the effect of the domain on the ATPase activity in native-like environment of saposin nanoparticles. HlyB∆CLD was reconstituted in LPC particles and its ATPase activity was measured in the presence and absence of unfolded or folded substrate (Fig. 6).

Based on the observed non-linear profile in presence of folded substrate, the data was fitted using the Michaelis-Menten model including substrate inhibition, suggesting a nearly two-fold stimulation of the basal ATPase activity. Adding unfolded substrate resulted in a 4-fold stimulation of the basal ATPase activity with a strong substrate inhibition at higher concentrations.

It is important to stress that the observed differences to HlyB are only due to the absence of the N-terminal CLD, as the assays were performed under identical conditions.

## Discussion

Type I secretion systems are ubiquitous amongst Gram-negative bacteria. Despite their high abundance and relatively simple build, the secretion mechanism is not yet fully understood.

HlyB is the ABC transporter that is part of the HlyA-type I secretion system in *E. coli*. Its purification and initial biochemical data in detergent solution have been reported^41^. Some ABC transporters contain additional accessory domains, which can be involved in substrate recognition and/or processing prior to transport. HlyB contains a CLD at its N-terminus, whose presence was shown to be essential for secretion *in vivo*. To study its influence on the interaction with the substrate, a HlyB derivative lacking this domain was created and termed HlyB∆CLD^41^.

In this study, we provide the first data for a functionally reconstituted ABC transporter in saposin-A lipoprotein nanoparticles^34^.

The use of an OmpF and AcrAB deficient C41(DE3)-strain resolved the problem of persistent impurities^41^. Furthermore, the protein was found to be stable and homogeneous in the non-ionic detergent DDM, whereas only LMNG has been previously observed to prevent the protein’s aggregation and precipitation (Fig. 1).

Even though purification was possible in DDM as well as in LMNG, reconstitution into saposin-lipoprotein nanoparticles was more efficient when employing DDM. A possible explanation might be a tight interaction of LMNG with the solubilised protein, which, combined with the detergent’s low cmc, might prevent the formation of homogeneous particles. This was indeed observed upon SEC, as the same reconstitution conditions in DDM and LMNG solutions produced homogeneous (DDM) and heterogeneous (LMNG) particles, respectively (Fig. 2).

Lyons *et al*. examined the reconstitution of saposin-A particles with a range of different phospholipids and natural extracts^38^. Reconstitution does not work when using *E. coli* total lipid extracts or pure phosphatidylethanolamine^39^, which makes up the largest portion of *E. coli* membranes^51^. Thus, to achieve the reconstitution of *E. coli* membrane proteins such as HlyB, the compromise of a non-native lipid environment had to be accepted.

DOPC is a commonly employed phospholipid for reconstitution experiments and it has been used before in combination with the saposin-A system^38^.

LPCs are present in natural membranes in low amounts where they have functions in regulation of cell responses or signalling^46,52,53^. They share the same head group with DOPC, but contain only one fatty acid tail. The bulky head group and the presence of only one fatty acid tail results in an “inverted cone-shape” for LPC^54,55^. In solution, LPC forms micelles and thus, can be considered as a detergent^46,56^.

Saposin-A has been shown to form bilayer-like structures with detergents in an otherwise detergent-free environment^37^. The crystal structure of saposin-A has been determined in the presence and absence of detergent molecules^37^. In this structure, saposin-A forms a small bilayer-like structure with detergents, that somewhat resemble a phospholipid bilayer^37^.

By using LPC as well as DOPC for the reconstitution of HlyB, we produced different environments around the membrane protein.

In this study, we have embedded the ABC transporter HlyB in detergent and lipid-derived saposin-particles, which enabled us to conduct functional studies with its dedicated substrate. Furthermore, the results highlight the importance of using systems that allow for studies in detergent-free buffers, since free detergent molecules may have a large impact on a membrane protein’s activity and/or functionality^26^; an effect that we also observed for HlyB (Fig. 3B).

Some minor adjustments in the amount of saposin-A used resulted in very pure and homogeneous protein preparations with highly reproducible ATPase activity. The separation of “full” and “empty” lipoprotein particles was performed by SEC. We conclude that the saposin-system is a seminal approach not only for structural, but also for the functional characterisation of membrane proteins.

In some buffer conditions, HlyB was inactive in detergent solution (not shown). However, reconstitution restored the basal ATPase activity of HlyB and HlyB∆CLD when using LPC or DOPC for reconstitution.

**Figure 7:**
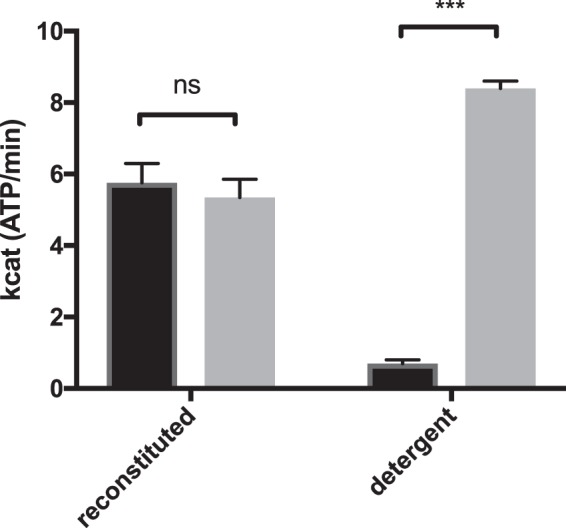
Comparison of the turnover numbers of HlyB (black) and HlyB∆CLD (grey) in detergent solution (data taken from Reimann *et al*. 2016^42^) and reconstituted in saposin-A nanoparticles (this publication). Error bars represent SEM of minimum two biological replicates. Ns :  not significant, ***p < 0.001.

Our results reveal the negative impact of free detergent on the activity of HlyB. Adding free LPC to DOPC-reconstituted HlyB significantly reduced its activity to approximately 10% (Fig. 3B). We assume that the solubilisation of the saposin-lipoprotein particles and the subsequent incorporation of HlyB into micelles, combined with high concentrations of free detergent are responsible for this effect. These results are in good agreement with studies performed with detergent-solubilised HlyB, where the determined v_max_ of HlyB corresponds to approximately 10% of the v_max_ determined in saposin-particles (Fig. 7)^41^.

In a previous study, it was observed that detergent-purified HlyB exhibits a maximum basal ATPase activity that is 10-fold lower than that of HlyB∆CLD, while the other kinetic parameters were not affected^41^ (Fig. 7). However, our results using membrane-reconstituted HlyB reveal different functional properties in native-like environment (Fig. 3A). The kinetic parameters of the basal ATPase activity, including the turnover number, were equal between HlyB and HlyB∆CLD within standards errors (Table 1, Fig. 7). The k_cat_ values obtained for both proteins are within the range of the k_cat_ of HlyB∆CLD measured in detergent solution^41^ but approximately 10-fold greater than that of HlyB in detergent solution (Fig. 7). The different behaviour in detergent solution and upon reconstitution has been described for other ABC transporters such as the maltose importer, P-gp (ABCB1) or MsbA^57–59^ and highlights the importance of the lipid environment for the ABC transporter.

As expected, both proteins show a strong cooperativity for ATP binding or hydrolysis, as it was described before for wildtype HlyB, HlyB∆CLD and isolated NBDs^18,41^.

The reconstituted H622A mutant did not show any hydrolytic activity (Fig. 3), a result described before for the isolated NBDs as well as detergent-purified HlyB^18,41^. Exchanging D551 to alanine in the isolated NBD of HlyB resulted in a loss of cooperativity and a 10-fold decrease in maximum enzyme velocity^50^. In the full-length protein, however, the mutation resulted in inactivation of the protein (Fig. 3), emphasising the possible different behaviour of isolated domains compared to fully assembled proteins.

We observed modulating effects of the substrate proHlyA when using LPC-reconstituted protein but not with DOPC (Figs 3, 4). A possible explanation might be the different packing characteristics of the bilayer when using LPC or DOPC, respectively. We suggest that the beforehand mentioned “inverted cone shape” of the LPC molecules^55,56^ result in a looser packing, which allows for interaction of HlyB with its substrate.

RTX proteins are secreted in an unfolded manner through type I secretion translocators^14,60^. Thus, it was assumed that the ABC transporter does not necessarily recognise folded proHlyA. However, previous measurements performed in detergent solution indicated an interaction of a folded C-terminal fragment, including the postulated secretion signal, with the ABC transporter. Our measurements in saposin-particles with folded full-length proHlyA confirmed the stimulatory effect, which was found to be independent of the presence of the CLD (Figs 5, 6). This does not necessarily reflect a physiologically relevant interaction since the substrate is only present in an unfolded state in the cytosol, and might also explain the overall relatively weak stimulation of about 60%. At higher substrate concentrations we observe an inhibition of the ATPase activities of HlyB and HlyB∆CLD (Figs 5, 6), which is a widespread phenomenon in enzyme kinetics^61^.

When adding unfolded proHlyA to the activity assay, a very weak stimulation was observed, while at higher concentration a substantial inhibition of the ATPase activity of HlyB dropping below the basal ATPase activity was observed (Fig. 5). We assume that the unfolded substrate is inserted into the transport channel of HlyB, but due to the lack of the other components of the secretion system the stalled HlyA locks the transporter resulting in inhibition of ATPase activity. The stimulatory interaction at low concentrations might be important for the proper orientation and/or insertion of the substrate.

In contrast, we observed a four-fold stimulation of the basal ATPase activity of HlyB∆CLD with unfolded proHlyA (Fig. 6). Thus, we suggest that the CLD is involved in placing HlyA into the translocation channel, sufficient to block the ATPase. In HlyB∆CLD, an inhibition of the ATPase can also be observed, but at higher substrate concentrations. This suggests the possible entry of the substrate to the translocation channel independently from the CLD, but whether these concentrations are physiologically relevant remains to be determined. Furthermore, a possible role of the CLD in preventing the substrate from aggregating needs to be considered.

## Summary

In our study, we show the interplay between reconstitution of an ABC transporter and its functionality. Furthermore, we demonstrated that the properties of a lipid bilayer can influence stimulation of an ABC transporter by its substrate, and that this is not necessarily reflected by a change in the basal ATPase activity. We established a protocol to embed the ABC transporter HlyB in detergent-derived lipoprotein particles and point out that the presence of free detergent micelles can affect the functionality of a membrane protein. Furthermore, we report functional assays for the first time with the 110 kDa full-length substrate.

## Supplementary information

Supplementary Information
