# Supplementary material for: Functional Reconstitution of HlyB, a Type I Secretion ABC Transporter, in Saposin-A Nanoparticles
Source: Sci Rep. 2019 Jun 10;9:8436. doi: 10.1038/s41598-019-44812-0 (PMC6558041; doi:10.1038/s41598-019-44812-0)
Supplement: Supplementary file 1 — Supplementary Information [file 41598_2019_44812_MOESM1_ESM.pdf]

Functional Reconstitution of HlyB, a Type I Secretion ABC Transporter, in Saposin-A Nanoparticles

Kerstin Kanonenberg, Sander HJ Smits, Lutz Schmitt*

Institute of Biochemistry, Heinrich Heine University, Universitaetsstr. 1, 40225 Duesseldorf, Germany

*Corresponding Author: Lutz Schmitt, Institute of Biochemistry, Universitaetsstrasse 1, 40225 Duesseldorf, Germany. Email: lutz.schmitt@hhu.de, phone: +492118110773.

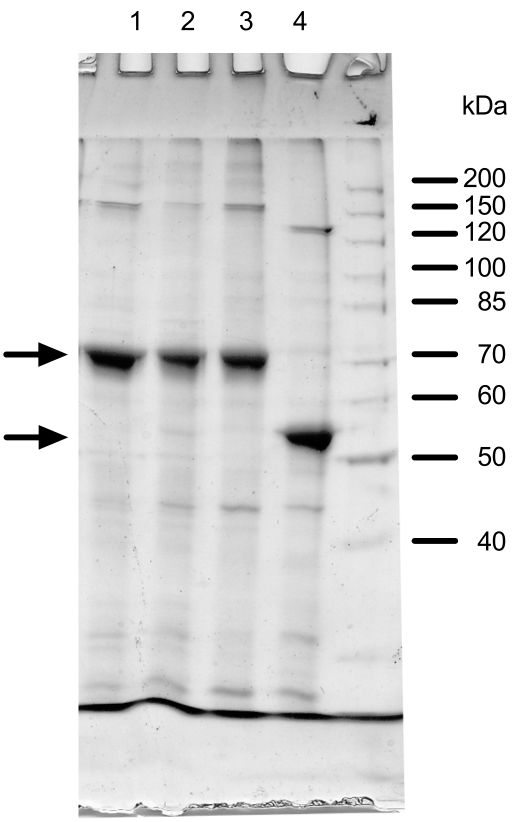

Supplementary Figure 1: Purification of HlyB. A – SDS-PAGE of purified HlyB (1), HlyB-H622A (2), HlyB-D551A (3) and HlyBΔCLD (4), stained with CBB. The arrows indicate the monomers of HlyB, HlyB H622A and HlyB D551A

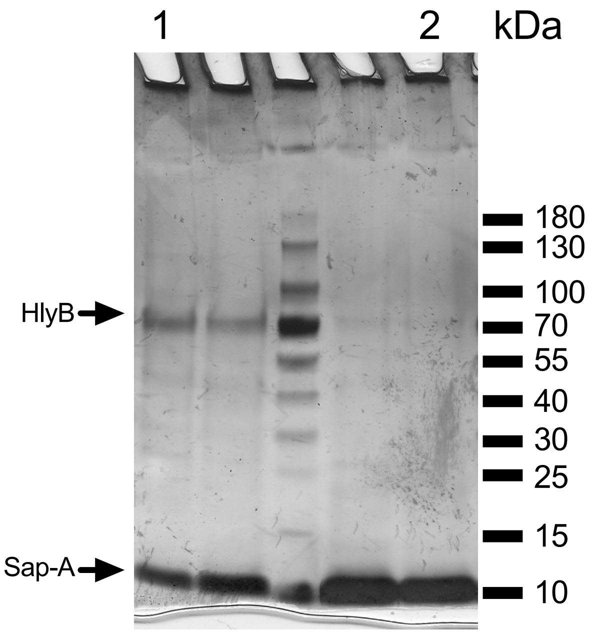

Supplementary Figure 2: SDS-PAGE of reconstituted HlyB (DDM) into LPC-particles after SEC on a 3-20% gradient silver-stained gel. Bands were visible for HlyB at 70 kDa and Saposin-A at 10 kDa (both indicated by arrows). (1) Peak at elution volume 15.5 mL (reconstituted HlyB), (2) peak at elution volume 17.8 mL ("empty" Saposin-A particles).

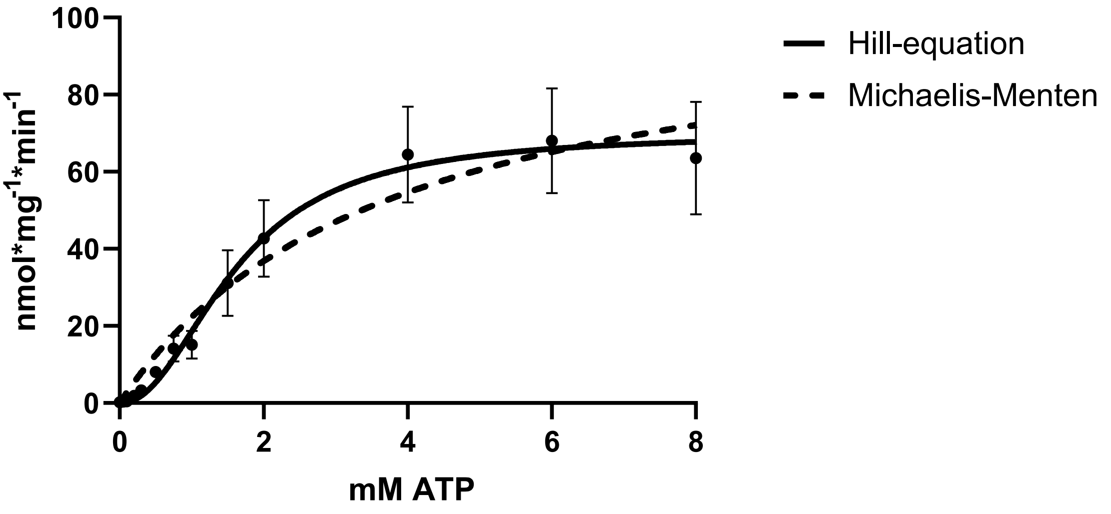

Supplementary Figure 3: Exemplary fit of the basal ATPase activity of HlyB (LPC-reconstituted) using the Hill-equation and the Michaelis-Menten model.

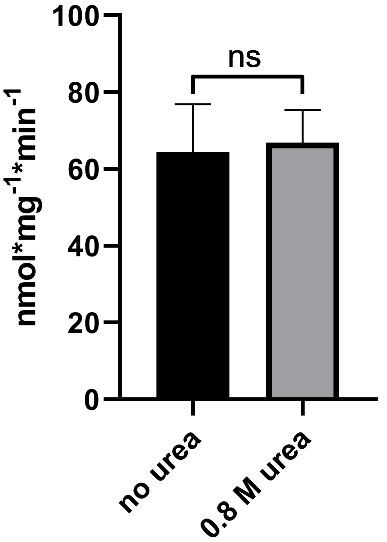

Supplementary Figure 4: Comparison of the basal ATPase activity of HlyB (LPC-reconstituted) in the presence and absence of 0.8 M urea. Significance was assessed using an unpaired t-test (ns: not significant, $p > 0.05$).

Supplementary Table 1: Results of the extra-sum-of-squares F-test to compare the goodness of fit of the Michaelis-Menten model and the Hill-equation to fit the basal ATPase activity of HlyB and derivatives. Based on this analysis, the Michaelis-Menten model was rejected in favour of the Hill-equation.

Curve	F-value	P-value
HlyB (LPC)	6.907	0.0122
HlyBΔCLD (LPC)	8.552	0.0072
HlyB (DOPC)	104.7	< 0.0001
HlyBΔCLD (DOPC)	15.29	0.0003
